# Dataset of optimized structures of reduced graphene-oxide with metal ions: Role of oxygen functional groups in metal ion adsorption

**DOI:** 10.1016/j.dib.2025.112161

**Published:** 2025-10-10

**Authors:** Michal Malček

**Affiliations:** Institute of Physical Chemistry and Chemical Physics, Faculty of Chemical and Food Technology, Slovak University of Technology in Bratislava, Radlinského 9, SK-812 37 Bratislava, Slovak Republic

**Keywords:** Adsorption, DFT, Reduced graphene oxide, Oxygen functional groups, Transition Metals

## Abstract

An interaction between a model system of reduced graphene oxide and a set of the first-row transition metal (TM) ions is investigated at the DFT level of theory. Optimized structures of the reduced graphene oxide with adsorbed TM ions in different spin states are presented. Three adsorption sites of the model system of reduced graphene oxide are considered, namely epoxide (O), hydroxyl (OH), and carboxyl (COOH) functional group. It is found that the energetically most favoured mode of interaction between the studied set of TM ions and the reduced graphene oxide is the adsorption on the OH group. In the case of Cr^2+^, Fe^2+^, Co^2+^, Ni^2+^, and Zn^2+^ ions, such interaction results in the cleavage of C—OH bond, consequently leading to formation of the TM-OH residue above the graphene basal plane. On the other hand, the energetically least favoured interaction between the TM ions and reduced graphene oxide occurs on the edge COOH groups. The optimized structures presented herein may also serve as a basis for reference in future computational studies of graphene oxide-based materials.

## Graphical abstract

**Figure:**
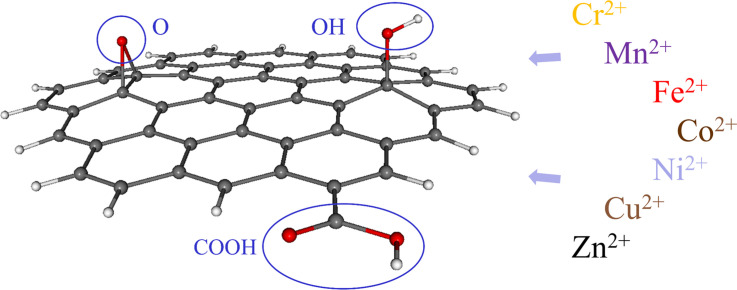

Specifications Table

**Table:** 

Subject	Engineering & Materials science
Specific subject area	*Graphene-based nanomaterials in environmental applications.*
Type of data	*Analyzed Tables and Images*
Data collection	*Data were calculated using HPC center at the Slovak University of Technology in Bratislava, which is a part of the Slovak Infrastructure of High-Performance Computing (SIVVP project, ITMS code 26230120002, funded by the European region development funds).*
Data source location	*Department of Physical Chemistry, Slovak University of Technology in Bratislava, Slovakia.*
Data accessibility	Repository name: Malcek, Michal (2025), “Optimized structures of reduced graphene-oxide with metal ions”, Mendeley Data, V1 Data identification number: DOI:10.17632/44tdcdyx68.1 Direct URL to data: https://data.mendeley.com/datasets/44tdcdyx68/1
Related research article	*None*

## 1 Value of the Data

• The presented dataset is composed of optimized structures of the finite size reduced graphene oxide with adsorbed transition metal ions, taking into account different metal ions in different spin states, and three adsorption sites for each metal ion were considered.
• The optimized geometries provided can be used as starting points for future studies dealing with metal particle adsorption on similar graphene oxide-based systems.
• Presented data can be also used as reference for theoretical calculations of various graphene-based materials, either graphene quantum dots (finite size) or infinite two-dimensional surfaces.
• Obtained results suggest that the graphene oxides possessing an increased amount of hydroxide functional groups (OH) are more suitable adsorbents of the first-row transition metal atoms. On the contrary, carboxyl functional groups (COOH) of reduced graphene oxide show the lowest affinity towards these metal ions.
• This dataset is relevant for researchers working in the field of theoretical calculations of graphene-based materials, surface science, and environmental applications (such as wastewater treatment).

## 2 Background

**Fig. 1:**
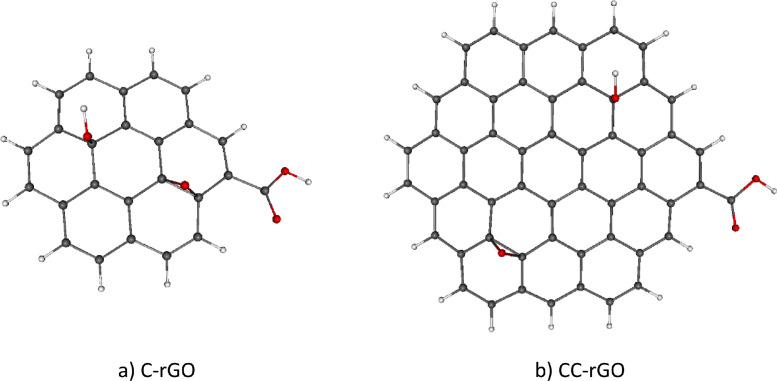
Optimized B3LYP-GD3/6–311G** structures of model rGO systems: coronene (C-rGO) and circumcoronene (CC-rGO) functionalized with hydroxyl (OH), epoxy (O), and carboxyl (COOH) groups. Colors of atoms: C – grey, H – white, O – red.

It is well-known that graphene oxide (GO) and its derivatives are promising adsorbents of transition metal (TM) ions, such as Co(II) [1], Cu(II) [2,3], Mn(II) [4], Zn(II) [5], Pb(II) [3,6], or Th(IV) [7], from aqueous solutions. Therefore, they can be utilized in environmental applications as materials capable of metal ions removal from wastewater [8,9]. The O-containing functional groups of GO represent the active sites for TM adsorption [2,7]. In the presented theoretical study, coronene (C_24_H_12_) and circumcoronene (C_54_H_18_), functionalized with epoxide (O), hydroxyl (OH), and carboxyl (COOH) functional groups, are used as model systems of the reduced graphene oxide (rGO) [10]. Circumcoronene [11] is often used in theoretical studies as finite-size model system of graphene surface due to its sufficient size and yet reasonable computational cost [[12], [13], [14], [15], [16], [17], [18]]. A smaller finite-size system, coronene [19] was selected for comparison purposes. It is assumed that the O and OH groups are located randomly at the graphene basal plane while COOH groups are usually present at its edges [[20], [21], [22], [23]]. Optimized structures of the model rGO systems, coronene (C-rGO) and circumcoronene (CC-rGO) are shown in Fig. 1. For the sake of simplicity, our model rGOs contain only three functional groups (see Fig. 1), enabling us to study the interaction between the particular functional group and the TM ion. The studied set of TM ions consists of Cr(II), Mn(II), Fe(II), Co(II), Ni(II), Cu(II), and Zn(II). The GO-based materials with adsorbed TM atoms possess increased reactivity when compared to parent GOs [24], hence, they can be further utilized in various industrial areas, for example as catalysts [24], energy storage devices [25] or biosensors [26].

## 3 Data Description

Calculated (B3LYP-GD3/6–311G**/PCM=H_2_O) optimized TM-O, TM-C, and O—C distances, TM Mulliken charges and spin populations, and reaction energies evaluated via Eq. (1) are shown in Tables 1 (CC-rGO) and 2 (C-rGO). These results point out that the OH group is an energetically preferred active site for adsorption of Cr^2+^, Ni^2+^, Cu^2+^, and Zn^2+^ ion, regardless of the size of model rGO system. Contrastingly, in the case of Mn^2+^, Fe^2+^, and Co^2+^ ions, the energetic preference of adsorption site varies with the size of rGO and the distribution of O-functional groups. The Mn^2+^ and Fe^2+^ ions are preferably adsorbed on the epoxide group (O) of CC-rGO (Table 1), while in the case of C-rGO, the adsorption on OH group is the energetically preferred one (Table 2). Interestingly, it is vice versa in the case of Co^2+^ ion: energetically preferred adsorption on OH group of CC-rGO and on O group of C-rGO. On the other hand, the carboxyl groups (COOH) show the lowest affinity to adsorb TM ions regardless of the studied rGO system. This result is in contradiction with some recent experimental studies pointing on involvement of COOH groups in the adsorption of Cu(II) and Pb(II) ions in aqueous solution/wastewater [2,3]. The reason of this disagreement can be the fact that rGOs with deprotonated COOH groups (possesing higher affinity towards TM ions) are not considered in the presented study.

**Table 1:** **CC-rGO:** Optimized B3LYP-GD3/6–311G**/PCM=H_2_O TM-O, TM-C and O—C distances (*d*, in Å) of the studied CC-rGO + TM ions in energetically preferred spin states (*M*_S_). Calculated Mulliken charges (*q*) and spins at the TM ions, as well as reaction energies (*E*_r_ in kJ mol^–1^) are also given. The energetically preferred binding site is highlighted in bold while the formation of TM-OH residue is indicated by blue color.

TM	Group	*M*_S_	*d* / Å			Mulliken / *e*		*E*_r_/ kJ mol^–1^
			TM-O	TM-C	O-C	*q*(TM)	*spin*(TM)	
**Cr^2+^**	O	4	1.88	3.19	1.44	1.68	3.99	–1861
	**OH**	**4**	1.86	3.72	3.11	1.62	4.00	–1947
	COOH	6	1.99			1.82	4.00	–1789
**Mn^2+^**	**O**	**5**	1.92	3.29	1.42	1.77	4.91	–1826
	OH	5	2.18	3.44	1.60	1.90	4.97	–1826
	COOH	7	2.13			1.90	4.97	–1781
**Fe^2+^**	**O**	**6**	1.85	3.11	1.43	1.70	3.85	–1902
	OH	8	1.82	5.04	2.99	1.63	3.86	–1835
	COOH	4	2.00			1.85	3.94	–1826
**Co^2+^**	O	3	2.05	3.55	1.50	1.87	2.96	–1911
	**OH**	**3**	1.82	3.64	3.06	1.62	2.85	–2059
	COOH	3	2.00			1.86	2.96	–1894
**Ni^2+^**	O	6	1.69	3.34	3.05	1.56	1.77	–1927
	**OH**	**2**	1.80	3.62	3.04	1.58	1.80	–2061
	COOH	2	1.97			1.81	1.92	–1897
**Cu^2+^**	O	1	2.33	2.04	1.46	1.13	0.00	–1957
	**OH**	**1**	2.11	2.06	1.51	1.14	0.00	–1980
	COOH	1	1.87			0.94	0.00	–1909
**Zn^2+^**	O	2	2.03	3.51	1.50	1.87	0.00	–1935
	**OH**	**2**	1.85	3.59	3.12	1.66	0.00	–2082
	COOH	2	1.97			1.86	0.00	–1922

**Table 2:** **C-rGO:** Optimized B3LYP-GD3/6–311G**/PCM=H_2_O TM-O, TM-C and O—C distances (*d*, in Å) of the studied C-rGO + TM ions in energetically preferred spin states (*M*_S_). Calculated Mulliken charges (*q*) and spins at the TM ions, as well as reaction energies (*E*_r_ in kJ mol^–1^) are also given. The energetically preferred binding site is highlighted in bold while the formation of TM-OH residue is indicated by blue color.

TM	Group	*M*_S_	*d* / Å			Mulliken / *e*		*E*_r_/ kJ mol^–1^
			TM-O	TM-C	O-C	*q*(TM)	*spin*(TM)	
**Cr^2+^**	O	6	2.15	3.65	1.52	1.87	4.00	–1717
	OH	4	1.81	3.58	2.61	1.86	3.65	–1808
	COOH	6	2.12			1.83	3.99	–1696
**Mn^2+^**	O	7	1.81	3.49	1.45	1.81	4.21	–1738
	OH	5	1.96	3.68	2.35	1.78	4.18	–1768
	COOH	7	2.13			1.90	4.97	–1729
**Fe^2+^**	O	4	2.11	3.43	1.48	1.88	3.97	–1794
	OH	6	1.81	3.70	2.71	1.88	4.07	–1836
	COOH	4	2.00			1.86	3.95	–1775
**Co^2+^**	O	3	1.84	3.29	1.43	1.69	2.86	–1895
	OH	5	2.05	3.54	1.60	1.85	2.97	–1877
	COOH	5	1.97			1.84	2.94	–1814
**Ni^2+^**	O	2	2.01	3.38	1.49	1.82	1.93	–1871
	OH	2	2.01	3.28	1.61	1.73	1.78	–1883
	COOH	2	1.96			1.78	1.89	–1845
**Cu^2+^**	O	1	2.54	2.05	1.45	1.15	0.00	–1889
	**OH**	**3**	1.95	3.06	1.53	1.36	0.61	–1925
	COOH	1	1.87			0.93	0.00	–1841
**Zn^2+^**	O	2	2.04	3.64	1.49	1.90	0.00	–1890
	**OH**	**2**	2.01	3.39	1.61	1.84	0.03	–1906
	COOH	2	1.96			1.86	0.00	–1871

As can be also seen from Table 1, Table 2, the most negative reaction energies, pointing on the highest affinity towards the TM ion, always correspond to the energetically preferred adsorption sites (and spin states). Similarly, the least negative reaction energies, corresponding to the lowest affinities towards the TM ions, are found for COOH group of rGO. Interestingly, upon the adsorption of Cr^2+^, Fe^2+^, Co^2+^, Ni^2+^, and Zn^2+^ on the OH group of CC-rGO, TM-OH residue is formed in a distance of approximately 3–4 Å above the CC-rGO backbone, see the values highlighted in blue color in Table 1. The formation of Co-OH, Ni-OH, and Zn-OH residues is connected with reaction energy exceeding –2000 kJ mol^–1^, see Table 1. Similarly, formation of TM-OH residue occurs upon the adsorption of Cr^2+^, Mn^2+^, and Fe^2+^ on the OH group of C-rGO. Herein, the calculated reaction energies are less negative (around –1800 kJ mol^–1^) than in the case of CC-rGO, see blue-colored values in Table 2. On the contrary, in the case of Mn^2+^ and Cu^2+^ adsorption on the OH group of CC-rGO, the OH group remains to be bound to the CC-rGO surface. Such different adsorption behavior of CC-rGO and C-rGO can be assigned not only to the size of model system but also to the distribution and positions of O-functional groups, i.e., the O and OH groups of C-rGO are closer to each other than the ones of CC-rGO, see Fig. 1. Naturally, the adsorption capacity of rGO is directly related to the number of O-functional groups (i.e. oxidation degree) [4]. Hence, to investigate this issue in more detail, further studies employing rGO systems with higher oxidation degree, such as ones in [27] or [28], are planned.

The adsorption of TM ions is connected with charge transfer of approximately 0.2–0.3 *e* from TM to the rGO surface, as can be seen from the Mulliken charges presented in Table 1, Table 2. The only exception is the Cu^2+^ adsorption, where Cu^2+^ is reduced to Cu^1+^, see the Mulliken charges of around 1.0 *e* in Table 1, Table 2. The calculated Mulliken charges of TM ions adsorbed on CC-rGO are by approximately 10 % lower than the ones of TM ions adsorbed on C-rGO. This finding suggests stronger adsorption of TM ions on larger CC-rGO system, which is confirmed also by shorter TM-O binding distances and more negative reaction energies, compare the data in Table 1, Table 2.

Last but not least, considering relatively similar values of calculated reaction energies compiled in Table 1, Table 2, one can conclude that the adsorption of particular TM ions on the studied rGOs is not selective. The selectivity of adsorption can be achieved, for example, via further functionalization of GO surfaces. Recently, Bakry et al. [29] reported a selective adsorption of Pb(II) ions on GO modified with 3-iminodiacetic acid while Li et al. [30] reported a selective adsorption of Cu(II) ions on rGO functionalized with triazole compounds.

**Fig. 2:**
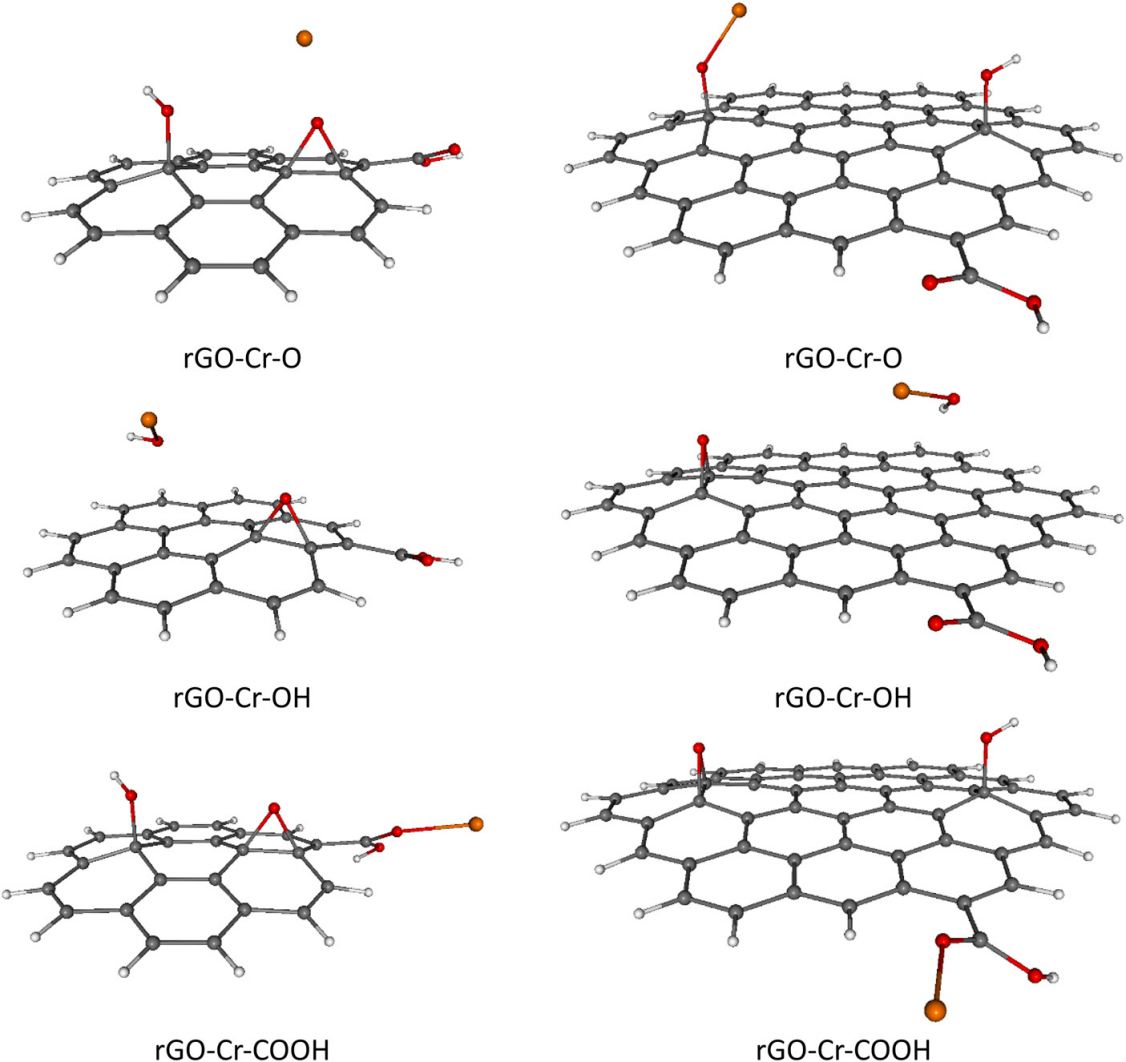
Optimized B3LYP-GD3/6–311G**/PCM=H_2_O rGO with Cr^2+^ ions adsorbed on different O-groups. Colors of atoms: C – grey, H – white, O – small red, Cr – orange. Left column - C-rGO, right column - CC-rGO.

**Fig. 3:**
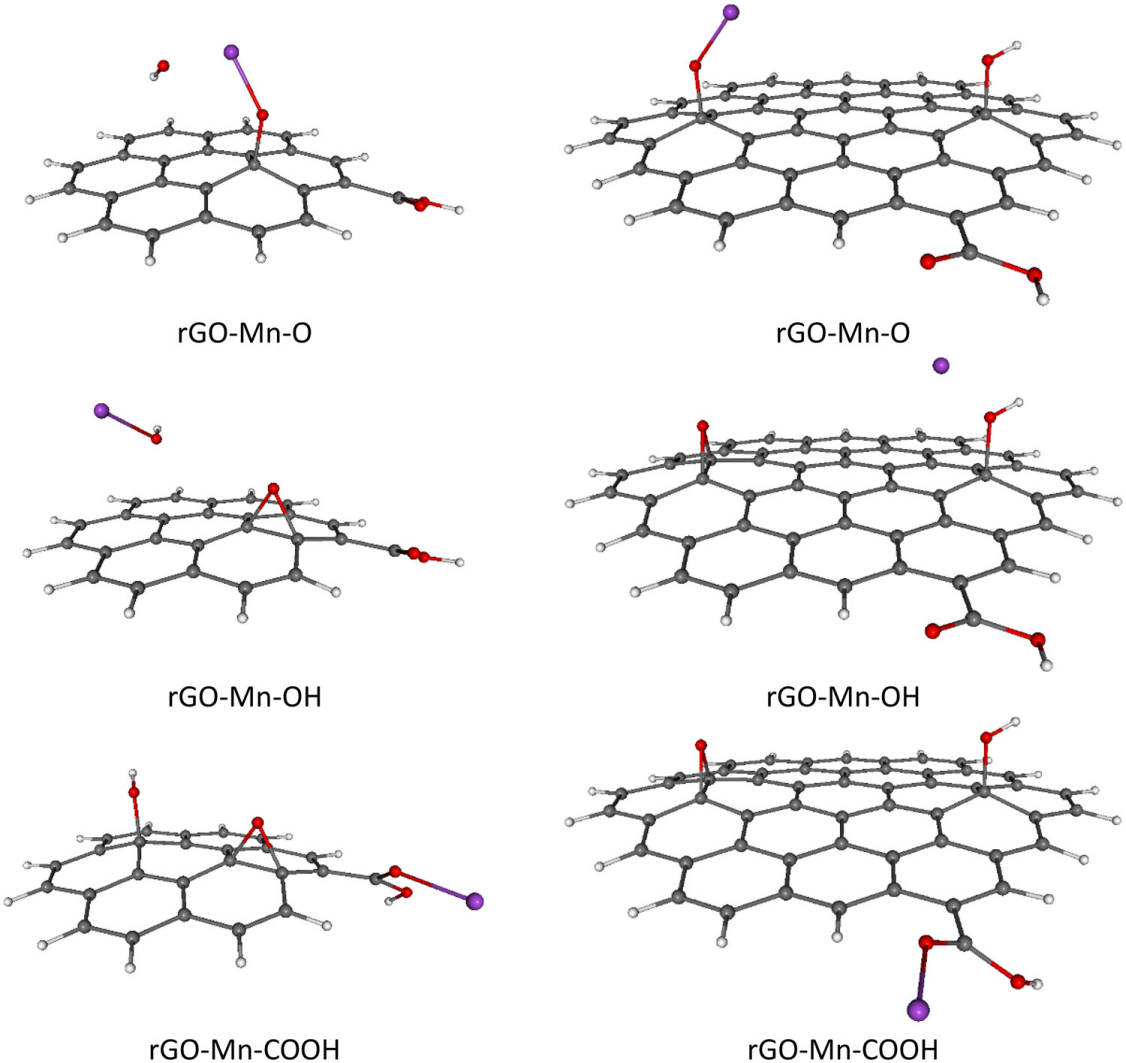
Optimized B3LYP-GD3/6–311G**/PCM=H_2_O rGO with Mn^2+^ ions adsorbed on different O-groups. Colors of atoms: C – grey, H – white, O – small red, Mn – purple. Left column - C-rGO, right column - CC-rGO.

**Fig. 4:**
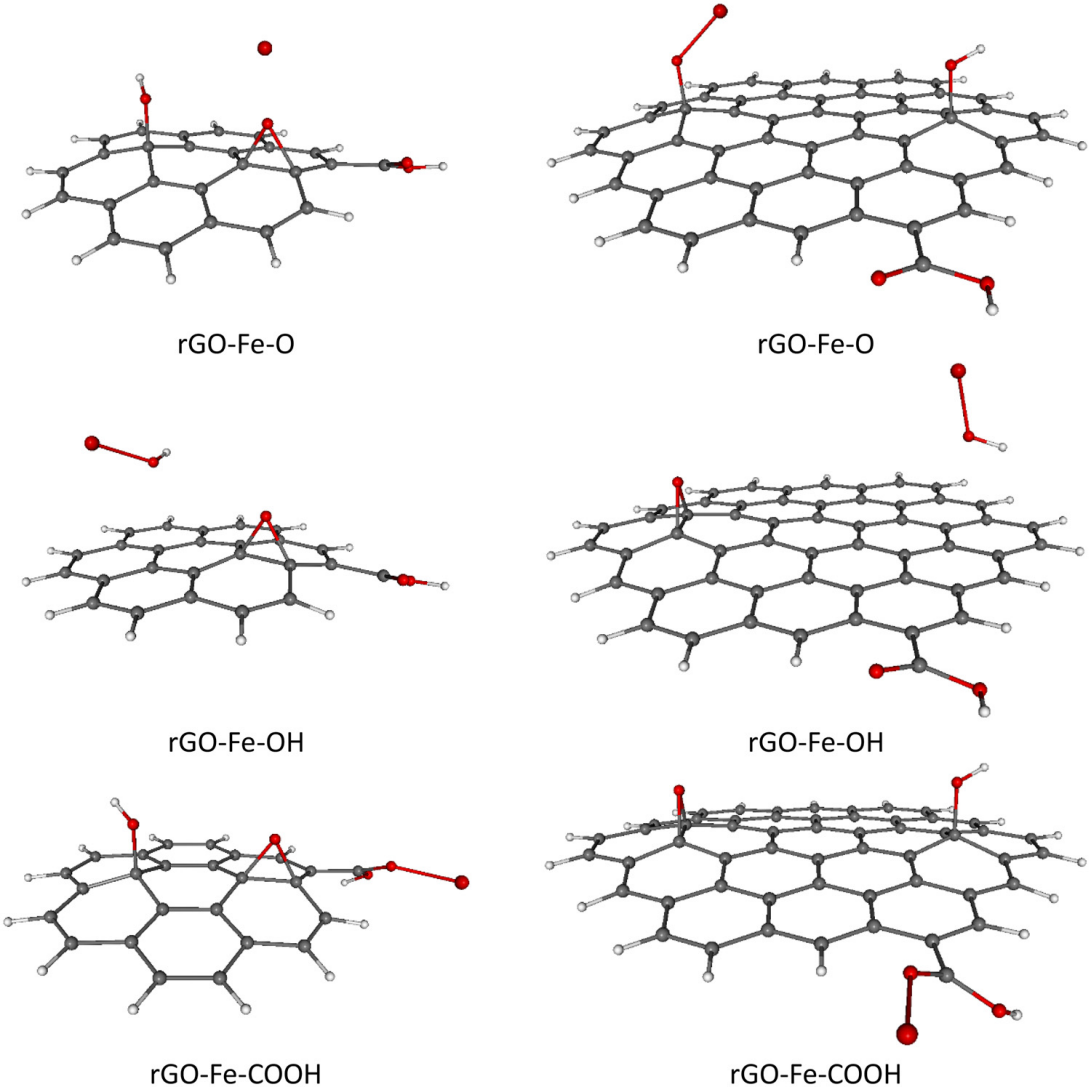
Optimized B3LYP-GD3/6–311G**/PCM=H_2_O rGO with Fe^2+^ ions adsorbed on different O-groups. Colors of atoms: C – grey, H – white, O – small red, Fe – large red. Left column - C-rGO, right column - CC-rGO.

**Fig. 5:**
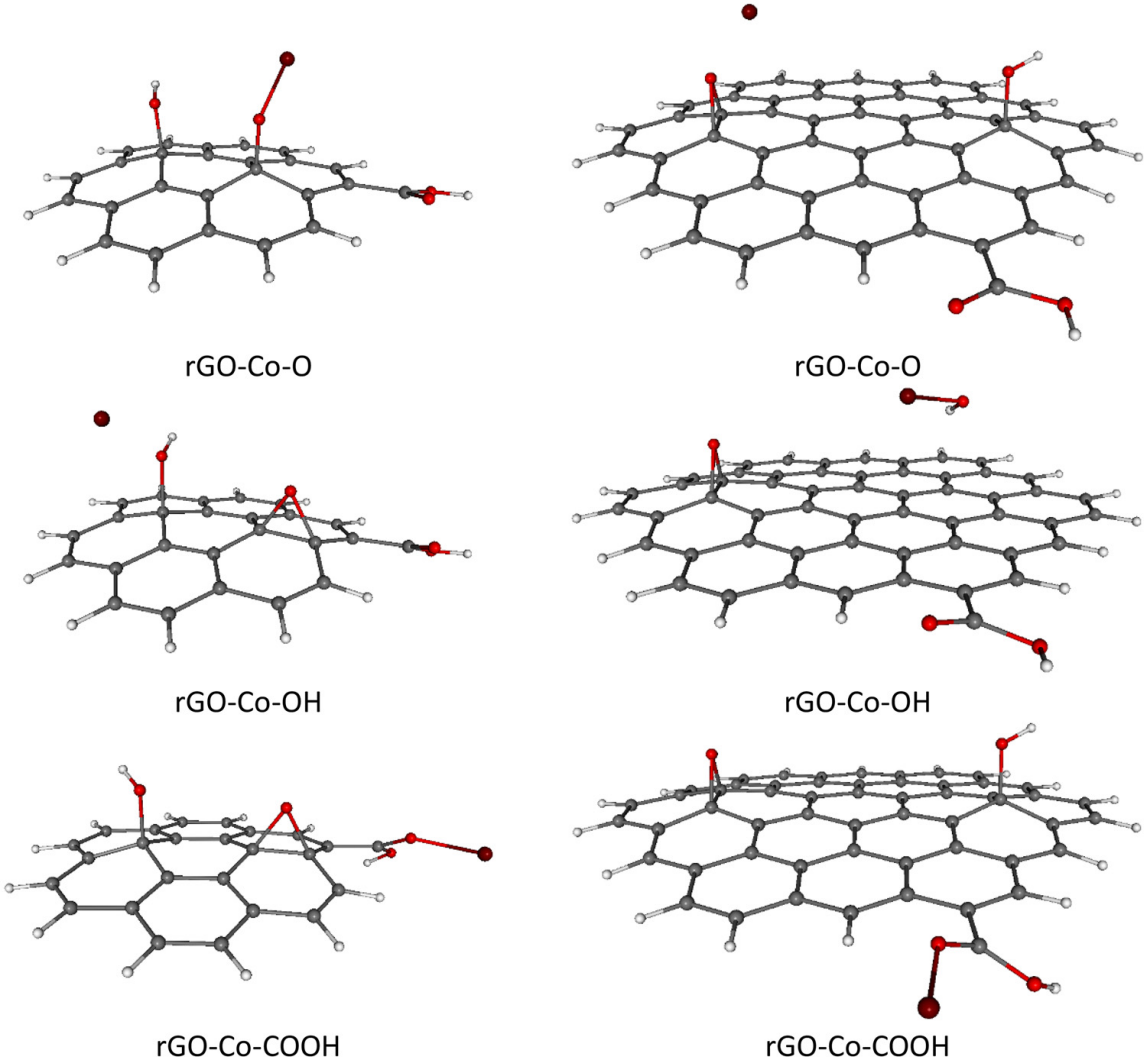
Optimized B3LYP-GD3/6–311G**/PCM=H_2_O rGO with Co^2+^ ions adsorbed on different O-groups. Colors of atoms: C – grey, H – white, O – small red, Co – wine. Left column - C-rGO, right column - CC-rGO.

**Fig. 6:**
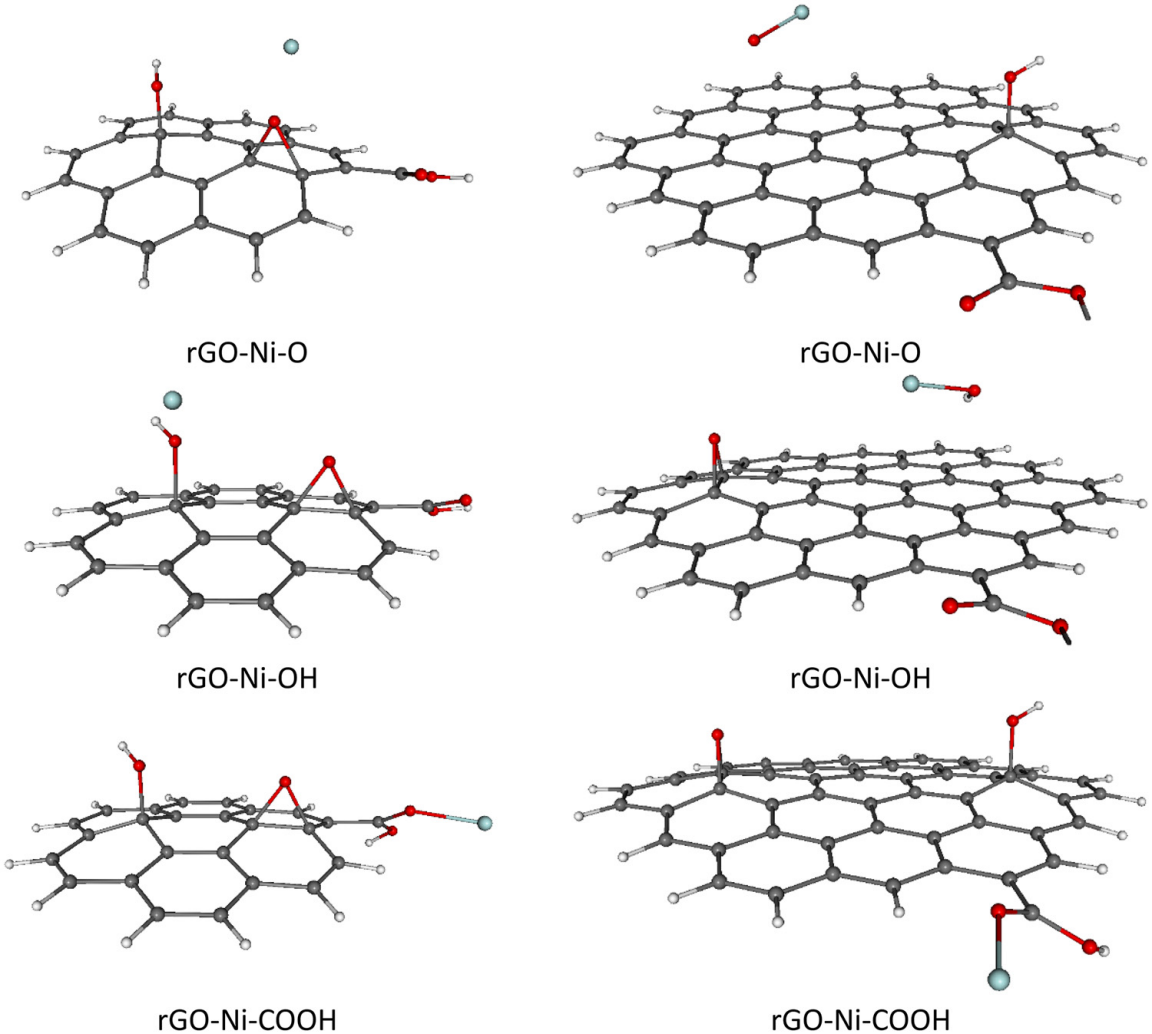
Optimized B3LYP-GD3/6–311G**/PCM=H_2_O rGO with Ni^2+^ ions adsorbed on different O-groups. Colors of atoms: C – grey, H – white, O – small red, Ni – cyan. Left column - C-rGO, right column - CC-rGO.

**Fig. 7:**
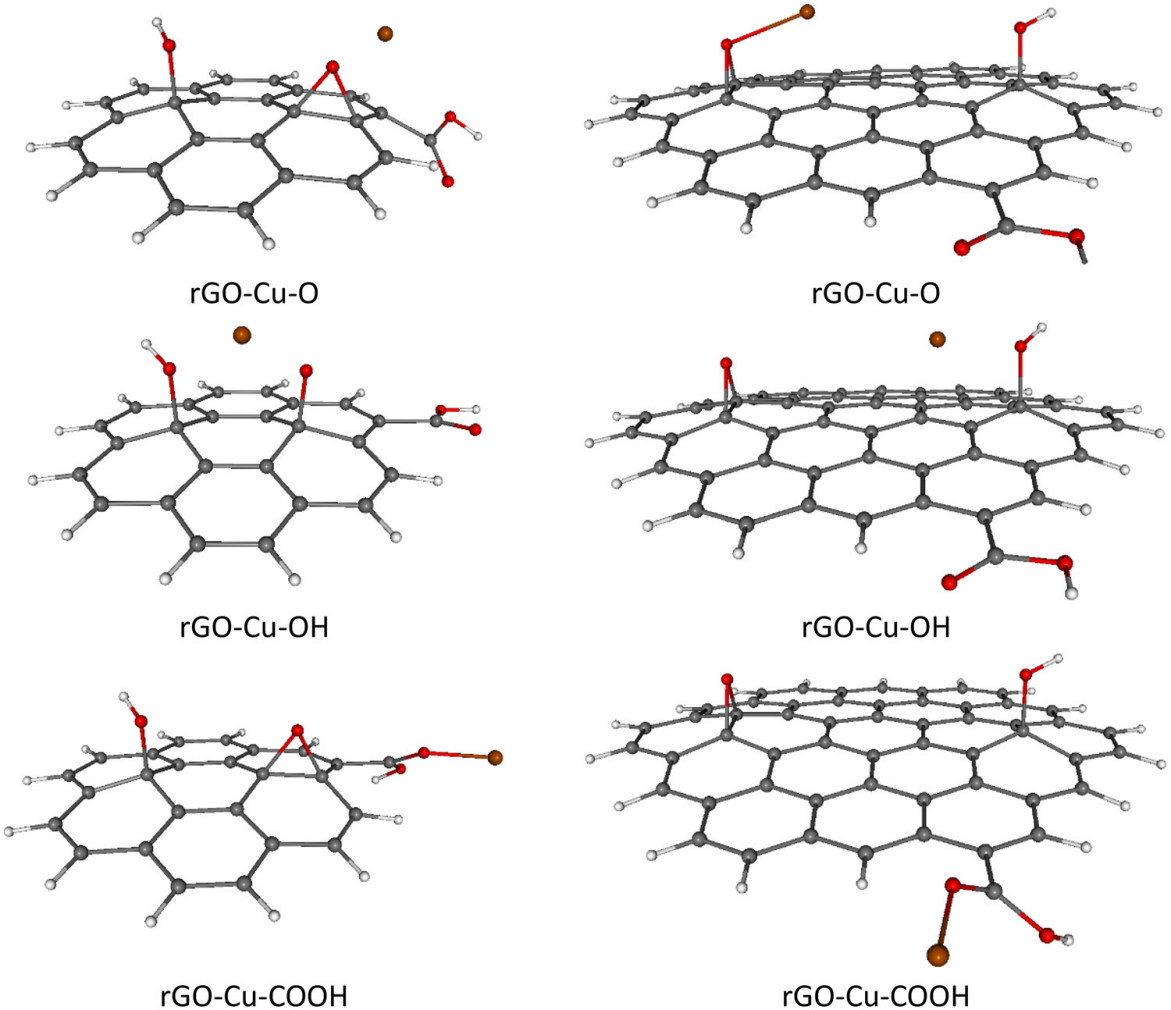
Optimized B3LYP-GD3/6–311G**/PCM=H_2_O rGO with Cu^2+^ ions adsorbed on different O-groups. Colors of atoms: C – grey, H – white, O – small red, Cu – brown. Left column - C-rGO, right column - CC-rGO.

**Fig. 8:**
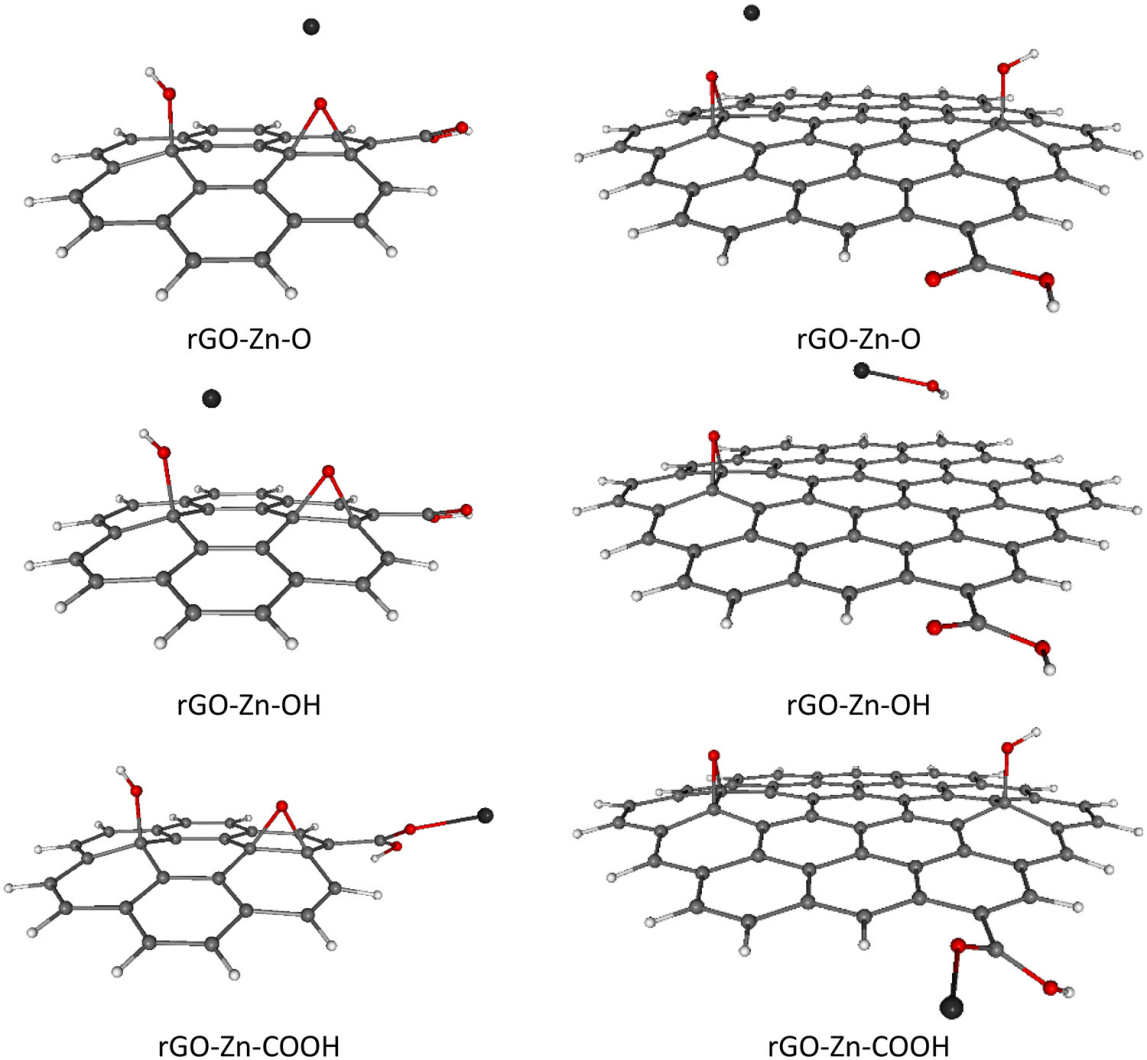
Optimized B3LYP-GD3/6–311G**/PCM=H_2_O rGO with Zn^2+^ ions adsorbed on different O-groups. Colors of atoms: C – grey, H – white, O – small red, Zn – dark grey. Left column - C-rGO, right column - CC-rGO.

For completeness, total and relative SCF energies of all studied rGO systems with adsorbed TM ions in different spin states are presented in Table 3, Table 4, including the corresponding Boltzmann population (BP) ratios evaluated at room temperature (*T* = 298.15 K).These results are in mutual agreement with the data shown in Table 1, Table 2. Naturally, the energetically preferred spin states of rGOs with heavier TM ions adsorbed (Ni^2+^, Cu^2+^, Zn^2+^) are mostly singlets or doublets, while in the case of lighter TM ions adsorption (Cr^2+^, Mn^2+^, Fe^2+^) the higher spin states such as quartet, pentet or sextet are energetically favored, see Table 3, Table 4. Optimized structures of all studied rGOs with TM ions adsorbed on each active site, in their energetically preferred spin states, are compiled in Fig. 2, Fig. 3, Fig. 4, Fig. 5, Fig. 6, Fig. 7, Fig. 8. The optimized geometries of these systems are also provided as xyz files in the Supplementary Material. Finally, the calculated total and relative energies of the single TM ions under study in different spin states are presented in Table 5, including the corresponding BP ratios.

**Table 3:** Spin state preference of the studied systems: total SCF energies (*E,* in atomic units), relative energies (Δ*E,* in kJ mol^–1^) and corresponding Boltzmann populations (BP, in %) of the rGO + TM ions (Cr^2+^, Mn^2+^, Fe^2+^) in different spin states (*M*_S_). The most stable spin state is highlighted in bold.

TM	Group	*M*_S_	System=CC-rGO			System=*C*-rGO		
			SCF *E*/ a.u.	Δ*E*/ kJ mol^–1^	BP / %	SCF *E*/ a.u.	Δ*E*/ kJ mol^–1^	BP / %
**Cr^2+^**	O	2	–3453.211338	143.5	0	–2305.859435	190.8	0
		4	–3453.233386	85.6	0	–2305.859269	191.2	0
		6	–3453.233122	86.3	0	–2305.897419	91.1	0
	OH	2	–3453.206656	155.7	0	–2305.885492	122.4	0
		**4**	**–3453.265976**	**0.0**	**52**	**–2305.932097**	**0.0**	**100**
		6	–3453.265901	0.2	48	–2305.908269	62.6	0
	COOH	2	–3453.125575	368.6	0	–2305.853726	205.8	0
		4	–3453.125826	368.0	0	–2305.853602	206.1	0
		6	–3453.205921	157.7	0	–2305.889344	112.3	0
**Mn^2+^**	O	1	–3559.687901	251.8	0	–2412.382308	257.8	0
		3	–3559.762619	55.6	0	–2412.396956	219.4	0
		**5**	**–3559.783797**	**0.0**	**28**	–2412.412378	178.9	0
		7	–3559.783756	0.1	27	–2412.469153	29.8	0
	OH	1	–3559.664926	312.1	0	–2412.334008	384.6	0
		3	–3559.740041	114.9	0	–2412.400763	209.4	0
		5	–3559.783683	0.3	25	**–2412.480501**	**0.0**	**96**
		7	–3559.783499	0.8	20	–2412.477580	7.7	4
	COOH	1	–3559.646791	359.7	0	–2412.246494	614.4	0
		3	–3559.666798	307.2	0	–2412.358763	319.6	0
		5	–3559.666760	307.3	0	–2412.358421	320.5	0
		7	–3559.766617	45.1	0	–2412.465910	38.3	0
**Fe^2+^**	O	2	–3672.431538	59.3	0	–2525.077196	185.2	0
		4	–3672.454014	0.3	47	–2525.131669	42.2	0
		**6**	**–3672.454132**	**0.0**	**53**	–2525.131494	42.3	0
		8	–3672.431319	59.9	0	–2525.091351	148.1	0
	OH	2	–3672.427855	69.0	0	–2525.093468	142.5	0
		4	–3672.427816	69.1	0	–2525.095674	136.7	0
		6	–3672.375521	206.4	0	**–2525.147745**	**0.0**	**100**
		8	–3672.428590	67.1	0	–2525.070151	203.7	0
	COOH	2	–3672.370662	219.2	0	–2525.053398	247.7	0
		4	–3672.425173	76.0	0	–2525.124452	61.2	0
		6	–3672.424804	77.0	0	–2525.124287	61.6	0
		8	–3672.382178	188.9	0	–2525.044419	271.3	0

**Table 4:** Spin state preference of the studied systems: total SCF energies (*E,* in atomic units), relative energies (Δ*E,* in kJ mol^–1^) and corresponding Boltzmann populations (BP, in %) of the rGO + TM ions (Co^2+^, Ni^2+^, Cu^2+^, Zn^2+^) in different spin states (*M*_S_). The most stable spin state is highlighted in bold.

TM	Group	*M*_S_	System=CC-rGO			System=*C*-rGO		
			SCF E/ a.u.	Δ*E*/ kJ mol^–1^	BP / %	SCF E/ a.u.	Δ*E*/ kJ mol^–1^	BP / %
**Co^2+^**	O	1	–3791.397009	314.3	0	–2644.097965	197.6	0
		3	–3791.460530	147.6	0	**–2644.173236**	**0.0**	**51**
		5	–3791.460504	147.6	0	–2644.173205	0.1	49
		7	–3791.459463	150.4	0	–2644.117790	145.6	0
	OH	1	–3791.464734	136.5	0	–2644.078881	247.7	0
		**3**	**–3791.516735**	**0.0**	**50.5**	–2644.158072	39.8	0
		5	–3791.516717	0.05	49.5	–2644.166528	17.6	0
		7	–3791.464726	136.5	0	–2644.080693	243.0	0
	COOH	1	–3791.411532	276.2	0	–2644.025724	387.3	0
		3	–3791.454128	164.4	0	–2644.108125	171.0	0
		5	–3791.453978	164.8	0	–2644.142312	81.1	0
		7	–3791.411392	276.6	0	–2644.063191	288.9	0
**Ni^2+^**	O	2	–3916.969172	165.9	0	–2769.678798	11.8	0.6
		4	–3916.967112	171.3	0	–2769.678631	12.2	0.4
		6	–3916.981178	134.4	0	–2769.633607	130.4	0
	OH	**2**	**–3917.032375**	**0.0**	**50.3**	**–2769.683290**	**0.0**	**70**
		4	–3917.032362	0.03	49.7	–2769.682451	2.2	29
		6	–3916.980390	136.5	0	–2769.611168	189.4	0
	COOH	2	–3916.969880	164.1	0	–2769.668886	37.8	0
		4	–3916.969422	165.3	0	–2769.668654	38.4	0
		6	–3916.927014	276.6	0	–2769.574046	286.8	0
**Cu^2+^**	O	1	–4049.129451	22.9	0	–2901.822283	36.2	0
		3	–4049.119509	49.0	0	–2901.788026	126.1	0
		5	–4049.070854	176.8	0	–2901.750741	224.0	0
	OH	**1**	**–4049.138190**	**0.0**	**100**	–2901.825622	27.4	0
		3	–4049.129964	21.6	0	**–2901.836056**	**0.0**	**100**
		5	–4049.077918	158.2	0	–2901.807954	73.8	0
	COOH	1	–4049.111091	71.1	0	–2901.804228	83.6	0
		3	–4049.104394	88.7	0	–2901.771641	169.1	0
		5	–4049.048774	234.8	0	–2901.694243	372.3	0
**Zn^2+^**	O	2	–4188.038282	146.8	0	–3040.739944	16.2	0.1
		4	–4188.036800	150.7	0	–3040.671308	196.4	0
	OH	**2**	**–4188.094195**	**0.0**	**100**	**–3040.746115**	**0.0**	**99.9**
		4	–4188.042330	136.2	0	–3040.671677	195.4	0
	COOH	2	–4188.033174	160.2	0	–3040.732655	35.3	0
		4	–4187.990630	271.9	0	–3040.652355	246.2	0

**Table 5:** Spin state preference of the studied TM ions: total SCF energies (*E,* in atomic units), relative energies (Δ*E,* in kJ mol^–1^) and corresponding Boltzmann populations (BP, in %) of the TM ions in different spin states (*M*_S_). The most stable spin state is highlighted in bold.

TM	*M*_S_	SCF *E*/a.u.	Δ*E*/ kJ mol^–1^	BP / %
**Cr^2+^**	1	–1043.411651	333.0	0
	3	–1043.485906	138.0	0
	**5**	–1043.538471	**0.0**	**100**
**Mn^2+^**	2	–1149.905774	516.1	0
	4	–1149.984181	310.3	0
	**6**	–1150.102359	**0.0**	**100**
**Fe^2+^**	1	–1262.598603	380.9	0
	3	–1262.651177	242.9	0
	**5**	–1262.743687	**0.0**	**100**
	7	–1262.579528	431.0	0
**Co^2+^**	2	–1381.662227	221.5	0
	**4**	–1381.746598	**0.0**	**100**
	6	–1381.527859	574.3	0
**Ni^2+^**	1	–1507.146447	301.3	0
	**3**	–1507.261206	**0.0**	**100**
	5	–1506.987708	718.1	0
**Cu^2+^**	**2**	–1639.397999	**0.0**	**100**
	4	–1639.097522	788.9	0
**Zn^2+^**	**1**	–1778.315121	**0.0**	**100**
	3	–1777.957667	938.5	0

## 4 Experimental Design, Materials and Methods

The studied systems were optimized using the B3LYP [[31], [32], [33], [34]] functional including the D3 version of Grimme's dispersion correction (GD3) [35,36] and 6–311G** basis set [37,38]. This computational protocol was used to ensure compatibility with our previous works [17,18,[39], [40], [41]]. The solvent effects of water were described using the polarizable continuum solvent model (PCM) [[42], [43], [44]] to mimic the metal ion adsorption in aqueous environment. The reaction energies *E*_r_ were calculated using the equation:

(1) $$E_{r}=E_{rGO-TM}\;-\;\left(E_{rGO}+E_{TM}\right)$$

where *E*_rGO-TM_ is total energy of the system with adsorbed TM ion, *E*_rGO_ and *E*_TM_ are energies of rGO and TM ion, respectively. BP ratios were evaluated at room temperature (298.15 K). All calculations were performed using Gaussian16 software [45]. Molekel software suite [46] was used for visualization of the optimized structures.

## Limitations

The limitations of presented study are as follows: i) the finite size model cannot fully compensate an infinite graphene sheet; ii) finite size graphene quantum dots of different sizes (for example circumpyrene, C_42_H_16_, or circumovalene, C_66_H_20_), and of different oxidation degree are not considered; iii) competitive adsorption of multiple transition metal ions is not considered; iv) effect of rGO interlayer stacking is not involved; v) performance of different DFT functionals and/or basis sets is not examined. All of these issues can be the subjects of further studies.

## Ethics Statement

The authors have read and follow the ethical requirements for publication in Data in Brief and confirm that the current work does not involve human subjects, animal experiments, or any data collected from social media platforms.

## CRediT authorship contribution statement

**Michal Malček:** Conceptualization, Methodology, Data curation, Writing – original draft, Investigation.

## Data Availability

Mendeley DataOptimized structures of reduced graphene-oxide with metal ions (Original data)

## Appendix Supplementary materials

Image, application 1

Image, application 2
